# Determinants of cessation of exclusive breastfeeding in Ankesha Guagusa Woreda, Awi Zone, Northwest Ethiopia: a cross-sectional study

**DOI:** 10.1186/1471-2393-14-262

**Published:** 2014-08-09

**Authors:** Tebikew Yeneabat, Tefera Belachew, Muluneh Haile

**Affiliations:** Department of Nursing and Midwifery, College of Medical and Health Sciences, Wollega University, PO.Box:395, Nekemte, Ethiopia; Department of Population and Family Health, College of Public Health and Medical Sciences, Jimma University, PO. Box:378, Jimma, Ethiopia; Department of Nursing, College of Public Health and Medical Sciences, Jimma University, PO.Box:378, Jimma, Ethiopia

**Keywords:** Exclusive breastfeeding, Cessation, Median duration, Ankesha Guagusa Woreda

## Abstract

**Background:**

Exclusive breast-feeding (EBF) is the practice of feeding only breast milk (including expressed breast milk) during the first six months and no other liquids and solid foods except medications. The time to cessation of exclusive breast-feeding, however, is different in different countries depending on different factors. Studies showed the risk of diarrhea morbidity and mortality is higher among none exclusive breast-feeding infants, common during starting other foods. However, there is no study that evaluated the time to cessation of exclusive breast-feeding in the study area. The aim of this study was to show time to cessation of EBF and its predictors among mothers of index infants less than twelve months old.

**Methods:**

We conducted a community-based cross-sectional study from February 13 to March 3, 2012 using both quantitative and qualitative methods. This study included a total of 592 mothers of index infant using multi-stage sampling method. Data were collected by using interviewer administered structured questionnaire. Bivariate and multivariate Cox regression analyses were performed.

**Results:**

Cessation of exclusive breast-feeding occurred in 392 (69.63%) cases. Among these, 224 (57.1%) happened before six months, while 145 (37.0%) and 23 (5.9%) occurred at six months and after six months of age of the index infant respectively. The median time for infants to stay on exclusive breast-feeding was 6.36 months in rural and 5.13 months in urban, and this difference was statistically significant on a Log rank (Cox-mantel) test. Maternal and paternal occupation, place of residence, postnatal counseling on exclusive breast-feeding, mode of delivery, and birth order of the index infant were significant predictors of cessation of exclusive breast-feeding.

**Conclusion:**

Providing postnatal care counseling on EBF, routine follow-up and support of those mothers having infants stressing for working mothers can bring about implementation of national strategy on infant and young child feeding.

## Background

According to World Health Organization and Ethiopian national strategy for infant and young child feeding, exclusive breast-feeding (EBF) is the practice of feeding only breast milk (including expressed breast milk) during the first six months and no other liquids and solid foods except medications [1, 2].

The WHO recommends EBF in the first six months beginning from the first hour of life. The global strategy on IYCF practices indicates that promoting optimal breast-feeding could prevent 13%, while those promoting optimal complementary feeding could prevent 6% of deaths in countries with high mortality rates [3, 4].

Studies showed prolonged breast-feeding benefits both for mother and infant health. It is the leading preventive child survival intervention. Nearly two million lives could be saved each year through six months of EBF and continued breast-feeding with proper complementary feeding for up to two years or longer. Maternal benefits include: reducing the risk of breast and ovarian cancer, reducing bleeding, preventing anemia by helping the uterus to return to its normal size and decreasing risks of new pregnancies by delaying the return of fertility [5–10]. Both too early and late introduction of breast-milk substitutes increases prevalence of under nutrition between 6–24 months. Offering foods to infants before six months reduces breast milk intake and interferes with full absorption of nutrients in the breast-milk. Complementary foods given to infants during the first six months can expose infants to infectious diseases, negatively impacting on their growth and development. As a result, none exclusive breast-fed infants are six times more likely to die because of diarrhea or respiratory infections than babies who do [11].

Infants are particularly vulnerable during the transition period when complementary feeding begins. The problem of malnutrition begins early in life, primarily during the first 12 months when growth faltering takes hold due to sub-optimal infant feeding practices [2, 12, 13]. Studies showed that breast-feeding is a time-dependent practice influenced by different factors, making initiation and termination of EBF different among lactating mothers across countries of the world [14, 15]. However, there is no study on predictors of early cessation of EBF in the study area in particular. This study identified the time to cessation of EBF and its predictors.

## Methods

### Study setting and sample

The study design was community based cross-sectional carried in Ankesha Guagusa Woreda in Awi Administrative Zone of Amhara Region, Ethiopia. Both quantitative and qualitative methods were used to collect data from February 13 to March 3, 2012 through face to face interview of mothers of index infant less than 12 months old. Epi Info 3.4.3 was used to calculate sample size using the two population proportion formula for urban and rural residences. The proportion of cessation of EBF in urban and rural areas 50% with hazard ratio (HR) of 2 for cessation of EBF in urban to rural were taken [14]. The value of Z = 1.96 at ***α***=0.05 and ***β***=0.2 were used. Equal size was allocated for urban and rural. Final sample size was 592 after adding 5% for none response and multiplying the sample by a design effect of 2 for the multi-stage sampling. Kebeles were first stratified into urban and rural areas. Then, three rural Kebeles and one urban Kebele were selected randomly to get the primary sampling unit. The sample size was allocated to urban and rural Kebeles, proportional to the size of their population. Then, mothers of index infant less than 12 months old were selected using simple random sampling.

Four focus group discussions (FGDs) (two from urban and two from rural consisting of eight participants in each group) conducted for qualitative data. Convenient sampling method was used to select participants of FGD from the Kebeles not included in the quantitative study having the same socio-demographic characteristics with in the Woreda.

### Measurements

Questionnaires for quantitative data were adapted from Ethiopian Demographic and Health Survey 2005, and from the tool used in Jimma Longitudinal Family Survey of Youth [16, 17]. Guidelines for qualitative data were developed after literature review. Questionnaires were first translated from English to “Amharic” and “Agewugna” and then back to English. Pre-test was done on 5% of the sample in an urban Kebele and a rural setting with similar status to the study community. Internal consistency of the items in the scales was checked, and all had good consistency (Cronbach’s alpha > 0.7). Quantitative data were collected by 10^th^ grade and above completed interviewers who were given two days intensive training on the tools and methods of data collection. Qualitative data were collected by the principal investigator.

### Data processing and analysis

Data on socio-demographic characteristics, obstetric and gynecological, food insecurity status, and current breast-feeding status were collected from the mothers having index child less than 12 months. The duration of EBF was the child’s age in months when consumption of other food (s) began. Exclusive breast-fed children during the time of interview were considered as censored cases and the duration of EBF was considered equal to child’s age.

After completion of editing, coding, and cleaning, data were entered to EpiData version 3.1 with double entry verification. Analysis was done using SPSS for windows version 16.0. The median age of infants at termination of EBF was estimated. Survival analysis using Kaplan-Meier survival curve was done to assess survival status of EBF. The long rank test was used to assess presence of significant difference in survival status of EBF between rural and urban. The likelihood of giving complementary food at each month and the cumulative risk of giving complementary food were estimated by the life table survival analysis.

Both bivariate and multivariate Cox proportional hazards model were used to identify factors that affect duration of EBF. The effect of each variable on the duration of EBF was assessed using bivariate Cox proportional hazards model to give a measure of the effect of each variable on the specific probabilities of the duration of exclusive breast-feeding (hazard function) in the absence of the control for other variables. Those variables with P < 0.25 in bivariate Cox regression model were entered spontaneously in multivariate Cox proportional hazards model to measure the effect of each category of each variable on the hazard function, after adjusting for the effects of other variables (and their categories) included in the model. Variables with P < 0.05 in multivariate Cox regression analysis were identified as the determinant factors associated with cessation of EBF during infancy. To assess association of cessation of EBF with economic status, wealth index was calculated based on ownership of fixed assets. Ownership of each fixed asset was given a value one and non-ownership a value of zero. Principal component analysis was done with a factors loading based on the Eigen values > =1. The first factors were taken and rank ordered into five quintiles.

### Qualitative data analysis

Qualitative data were transcribed verbatim into English language and thematic areas were identified based on the objective of the study. Ideas were organized under the themes using an open color coding and presented in narratives using well-said verbatim of the study participants as illustrations. The results were triangulated with the quantitative finding.

### Ethical consideration

Formal written letter of Ethical approval was obtained from Jimma University Ethical Review Board and official letter of cooperation was obtained and secured to the selected Kebeles from Ankesha Guagusa Woreda Health Office. Informed verbal consent was secured from study participants after assuring about the confidentiality of the data. The purpose, potential risks and benefits of participating, and the right to with draw from the study at any time was explained to the study participants.

## Results

Out of 592 mother-infant pairs sampled, 563 were included in the analysis making the response rate 95.1%. The mean (±SD) age of mothers was 29.27 (±6.29) years and the mean (±SD) age of infants was 6.15 (±2.96) months. Ninety six point eight percent of the respondents were Orthodox Tewahido Christians followed by Protestants and Muslims each accounting for 1.6% (Table 1).

**Figure 1:**
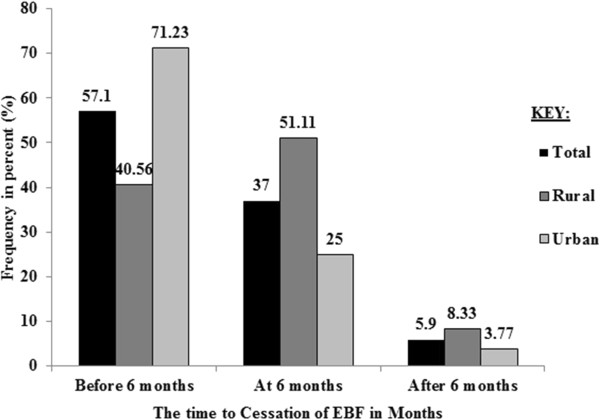
**Percentage of cessation of EBF by the duration of time, Ankesha Guagusa Woreda, Awi administrative zone, Northwest Ethiopia 2012.**

Cessation of EBF was happened in 392 cases out of which 212 and 180 were from urban and rural respectively. The remaining 171 cases were censored during the interview. Proportion of censored cases was more in rural 99 (35.5%) than urban 72 (25.4%). Among mothers who ceased to breast-feed exclusively, 57.1% occurred before six months, 37.0% occurred at six months and 5.9% occurred after six months of age of the index infant (Figure 1).

**Figure 2:**
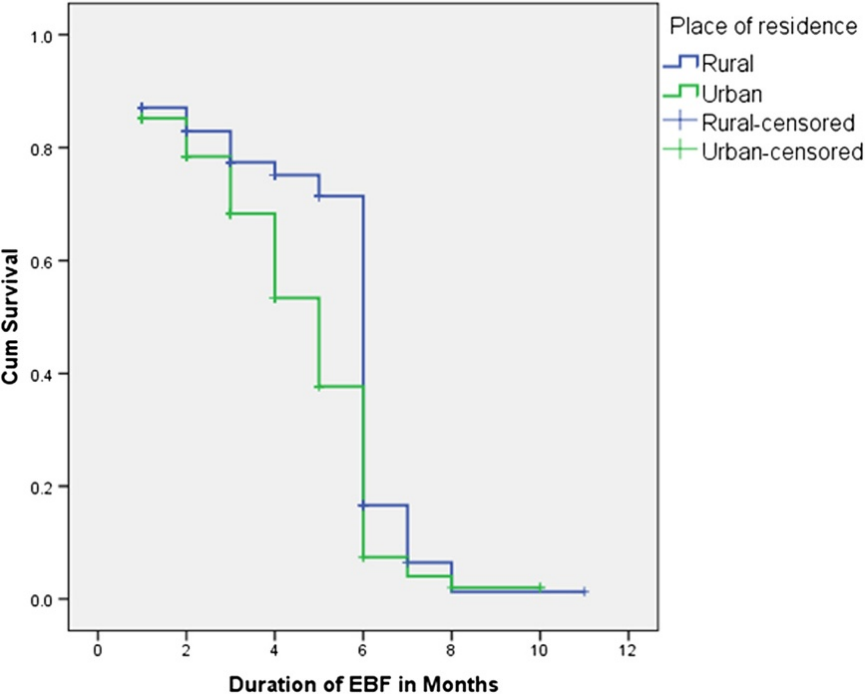
**Kaplan-Meier cumulative survival probability functions of EBF for rural and urban areas, Ankesha Guagusa Woreda, Awi administrative zone, Northwest Ethiopia 2012.**

**Figure 3:**
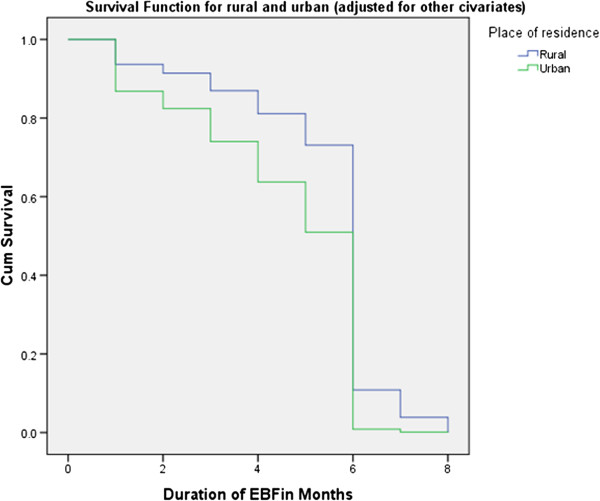
**Cumulative survival probability functions of exclusive breast feeding for rural and urban areas in adjusted multivariable cox proportional hazard model, Ankesha Guagusa Woreda, Awi administrative zone, Northwest Ethiopia 2012.**

The cumulative proportion of survival probability in life table indicated that the percentage of infants that remained on EBF for the first 5 months was 63%. Proportion of cessation of EBF before the age of six months was higher in urban than rural (Figures 2 and 3).

**Table 1:** **Socio-demographic characteristics of the respondents, Ankesha Guagusa Woreda, Awi administrative zone, Northwest Ethiopia 2012**

Variables		Place of residence		Total (n = 563) no. (%)
		Rural (n = 279) no (%)	Urban (n = 284) no. (%)	
Ethnicity	Agew	257 (92.1)	140 (49.3)	397 (70.5)
	Amhara	22 (7.9)	144 (50.7)	166 (29.5)
Educational status	No education	206 (73.8)	49 (17.3)	255 (45.3)
	Primary	56 (20.1)	91 (32.0)	147 (26.1)
	Secondary	15 (5.4)	68 (23.9)	83 (14.7)
	College and above	2 (0.7)	76 (26.8)	78 (13.9)
Marital status	Married	270 (96.8)	279 (98.2)	549 (97.5)
	Widowed	3 (1.1)	3(1.1)	6 (1.1)
	Divorced	6 (2.2)	2(0.7)	8 (1.4)
Occupation	Housewife	10 (3.6)	142 (50.0)	152 (27)
	Farmer	260 (93.2)	12 (4.2)	272 (48.3)
	Merchant	1 (0.4)	43 (15.1)	44 (7.8)
	Civil servant	2 (0.7)	69 (24.3)	71(12.6)
	Student	2 (0.7)	12 (4.2)	14 (2.5)
	Other	4 (1.4)	6 (2.1)	10 (1.8)
Sex of index child	Male	143 (51.3)	146 (51.4)	289 (51.3)
	Female	136 (48.7)	138 (48.6)	274 (48.7)

The median duration of EBF was estimated to be 6.06 months for the entire subjects, 6.36 months in rural and 5.13 months in urban. The cumulative survival probability of EBF for the first seven months was higher in rural compared to urban. Similarly Kaplan-Meir survival curve on Figure 3 illustrates that survival curve for rural residence in the first 6 months was constantly above the curve for urban residence. This difference was statistically significant on a Log rank (Cox-Mantel) test (P < 0.0001) (Table 2).

**Table 2:** **Cumulative survival probabilities and hazard rates of EBF at different ages of infants in months for rural and urban mothers having index infant less than one year, Ankesha Guagusa Woreda, Awi administrative zone, Northwest Ethiopia 2012**

Place	EBF interval (months)	Interval start time (months)	No. of cases entering this interval	No of terminal events	No. of censored cases	Proportion of surviving	Cumulative survival probability at the end of interval	Hazard rate
**Rural**	0-1	0	279	0	0	1.00	1.00	0.00
	1-2	1	279	36	14	0.87	0.87	0.14
	2-3	2	229	11	7	0.95	0.83	0.05
	3-4	3	211	14	27	0.93	0.77	0.07
	4-5	4	170	5	25	0.97	0.74	0.03
	5-6	5	140	7	13	0.95	0.70	0.05
	6-7	6	120	92	10	0.20	0.14	1.33
	7-8	7	18	11	2	0.35	0.05	0.96
	8-9	8	5	4	0	0.20	0.01	1.33
	9-10	9	1	0	0	1.00	0.01	0.00
	10-11	10	1	0	0	1.00	0.01	0.00
	11-12	11	1	0	1	1.00	0.01	0.00
**Urban**	0-1	0	284	0	0	1.00	1.00	0.00
	1-2	1	284	42	16	0.85	0.85	0.16
	2-3	2	226	18	14	0.92	0.78	0.09
	3-4	3	194	25	14	0.87	0.67	0.14
	4-5	4	155	34	12	0.77	0.52	0.26
	5-6	5	109	32	11	0.69	0.36	0.37
	6-7	6	66	53	2	0.18	0.07	1.38
	7-8	7	11	5	0	0.55	0.04	0.59
	8-9	8	6	3	1	0.45	0.02	0.75
	9-10	9	2	0	0	1.00	0.02	0.00
	10-11	10	2	0	2	1.00	0.02	0.00
**Total**	0-1	0	563	0	0	1.00	1.00	0.00
	1-2	1	563	78	30	0.86	0.86	0.15
	2-3	2	455	29	21	0.93	0.80	0.07
	3-4	3	405	39	41	0.90	0.72	0.11
	4-5	4	325	39	37	0.87	0.63	0.14
	5-6	5	249	39	24	0.84	0.53	0.18
	6-7	6	186	145	12	0.19	0.10	1.35
	7-8	7	29	16	2	0.43	0.04	0.80
	8-9	8	11	7	1	0.33	0.01	1.00
	9-10	9	3	0	0	1.00	0.01	0.00
	10-11	10	3	0	2	1.00	0.01	0.00
	11-12	11	1	0	1	1.00	0.01	0.00

The most common complementary foods given to children before the age of six months were: cow’s milk (73.7%), water/tea (61.6%), cereal based fluids such as porridge (39.7%) and others mainly fresh butter (24.6%). Fresh butter was the second most common food given for the infants at first month of age accounting 40 (51.3%) next to cow’s milk 45 (57.7%). The main reasons for introduction of complementary food before six months of age were: mother returned to work (30.8%) followed by little or no breast milk (25.89%). Sickness of the child was the main reason during the first month 42 (53.8%) followed by not enough milk 8 (10.3%).

Cessation of EBF was significantly associated with age of mother, educational status of mother and partner, occupation of the mother and partner, place of residence, provision of counseling on EBF during ANC, delivery place, mode of delivery, birth attendant, PNC utilization, provision of counseling on EBF during PNC, parity of the mother, birth order of the index infant and food insecurity. However, it was not significantly associated with wealth index, marital status, ANC visit and sex of the index infant.

Mothers below the age of 20′s were 1.5 times more likely to cease EBF early than those who were in second half of 30′s and above (HR = 1.504; 95% CI, 1.014-2.232; P < 0.05). Mothers with educational status of college and above were 2.34 times more likely to cease EBF early compared with none educated mothers (HR: 2.340; 95% CI, 1.765-3.103; P < 0.001). However, it was insignificant after adjustment for other covariates. The hazard of early cessation of EBF was 1.7 times more in mothers with their partner’s educational status is college and above compared to the mothers with none educated partner (HR = 1.698; 95% CI = 1.327-2.172; P < 0.001), but this was insignificant in adjusted Cox proportional hazards model. Mothers who were engaged in different jobs (including daily laborer, house servant and pottery) were 2.182 times more likely to terminate EBF early compared to housewife mothers (HR: 2.182; 95% CI = 1.007-4.731; P = 0.048) and civil servant mothers 1.74 times likely cease EBF early compared to housewives (HR = 1.742; 95% CI = 1.268-2.395; P = 0.001). However, the hazard of early cessation of EBF for farmer mothers was less compared to housewives (HR: 0.771; 95% CI = 0.601-0.990; P = 0.041). Statistical significance was retained in adjusted Cox proportional hazards model in which mothers who were engaged in different jobs (daily labor, house servant and pottery) were 5.156 times more likely to have early cessation of EBF compared to housewives (AHR: 5.156; 95% CI = 1.018-26.117; P = 0.048), the significance for other groups was attenuated after adjustment. Similarly, mothers with civil servant partner were 1.72 times more likely to terminate EBF early compared to mothers with farmer partner (HR: 1.718; 95% CI = 1.356-2.176; P < 0.001). However, the likely hazard was about 0.2 times for mothers with civil servant partner compared to mothers with farmer partner after adjustment in Cox proportional hazards model (AHR: 0.195; 95% CI = 0.043-0.893; P = 0.035). Mothers living in urban were 1.599 times more likely to cease EBF early compared to those living in rural (HR: 1.559; 95% CI = 1.276-1.905; P < 0.001). Statistical significance was also retained in adjusted Cox proportional hazards model in which hazard of early cessation of EBF was 2.175 times more in urban compared to rural mothers (AHR: 2.175; 95% CI = 1.054-4.489; P = 0.036) (Table 3).

**Table 3:** **Bivariate and multivariable cox proportional hazards model predicting the hazards of cessation of exclusive breastfeeding by socio-demographic characteristics of mothers of index infants in Ankesha Guagusa Woreda, Awi administrative zone, Northwest Ethiopia 2012**

Variables	Cessation of exclusive breastfeeding			
	CHR^*^(95% CI)	P	AHR^†^(95% CI)	P
**Age of the mother**				
15-20	1.504 (1.014-2.232)	0.043	1.133 (0.478-2.684)	0.777
21-25	1.059 (0.759-1.475)	0.737	0.937 (0.555-1.584)	0.809
26-30	1.036 (0.779-1.377)	0.809	0.703 (0.444-1.111)	0.131
31-35	0.918 (0.664-1.269)	0.605	0.894 (0.573-1.396)	0.623
36+	1		1	
**Education of mother**				
No education^‡^	1		1	
Primary^§^	1.108 (0.857-1.432)	0.433	1.028 (0.627-1.686)	0.914
Secondary^**^	1.623 (1.204-2.188)	0.001	1.004 (0.513-1.967)	0.990
College and above^††^	2.340 (1.765-3.103)	0.000	2.490 (0.920-6.739)	0.073
**Education of partner**				
No education	1		1	
Primary	0.938 (0.718-1.224)	0.637	1.016 (0.642-1.609)	0.945
Secondary	1.169 (0.831-1.643)	0.370	0.903 (0.462-1.766)	0.766
College and above	1.698 (1.327-2.172)	0.000	1.646 (0.717-3.780)	0.240
**Marital status**				
Currently married	1.106 (0.590-2.074)	0.753	--------	----
Single	1			
**Occupation of the mother**				
Housewife	1		1	
Farmer	0.771 (0.601-0.990)	0.041	0.481 (0.138-1.679)	0.251
Merchant	1.098 (0.739-1.631)	0.642	0.914 (0.471-1.774)	0.791
Civil servant	1.742 (1.268-2.395)	0.001	0.813 (0.345-1.917)	0.636
Student	1.516 (0.831-2.767)	0.175	1.529 (0.592-3.944)	0.380
Others^‡‡^	2.182 (1.007-4.731)	0.048	5.156 (1.018-26.117)	0.048
**Occupation of the partner**				
Farmer	1			
Merchant	1.392 (1.065-1.821)	0.016	0.390 (0.093-1.641)	0.199
Civil servant	1.718 (1.356-2.176)	0.000	0.195 (0.043-0.893)	0.035
Student	1.642 (0.407-6.620)	0.485	1.328 (0.478-2.684)	0.877
Others^§§^	1.272 (0.791-2.046)	0.321	0.412 (0.084-2.014)	0.274
**Place of residence**				
Rural	1		1	
Urban	1.559 (1.276-1.905)	0.000	2.175 (1.054-4.489)	0.036
**Wealth index**				
Poorest	0.863 (0.622-1.199)	0.380	1.295 (0.559-3.001)	0.546
Poorer	0.963 (0.699-1.327)	0.817	1.180 (0.520-2.677)	0.692
Middle	0.987 (0.725-1.344)	0.934	0.972 (0.571-1.652)	0.915
Richer	0.750 (0.549-1.025)	0.071	0.751 (0.469-1.202)	0.233
Richest	1		1	

*Crude Hazard Ratio.

†Adjusted Hazard Ratio.

‡Those who did not attended formal education.

§Those who had completed grade 1-8.

**Those who had completed grade 9-12.

††Those who had attended colleges including technique.

‡‡Daily labour, potter and house servant.

§§Daily labour, carpenter, building worker, weaver, smith and metal worker.

The hazards of early cessation of EBF was 1.4 times more in mothers who were not provided with antenatal counseling on EBF compared to those who were provided counseling on EBF during ANC (HR = 1.519; 95% CI = 1.186-1.945; P = 0.001). However, it was insignificant in adjusted Cox proportional hazards model. Surprisingly, the place of delivery of index infant was inversely associated with duration of EBF in which infants born in home had 0.67 times the hazard of early cessation of EBF compared to those who were born in health institution (HR = 0.670; 95% CI = 0.543-.826; P < 0.001). This inverse association might be attributed to the fact that early cessation of EBF was high in mothers who gave birth of their index infant by cesarean section (CS) than by vaginal delivery as revealed in Table 3. The risk of early cessation of EBF was 2.543 times more in those who gave birth by CS compared to vaginal delivery (HR = 2.543; 95% CI = 1.620-3.993; P < 0.001) and it was also significant on adjusted Cox proportional hazards model (AHR: 2.103; 95% CI = 1.173-3.770; P = 0.013). However, the significance was attenuated for the place of delivery in adjusted Cox proportional hazards model. The risk of early cessation of EBF was 0.7 times in mothers who were assisted with none trained traditional birth attendants (NTTBA) compared to mothers who were assisted by relatives/friends/families (HR = 0.703; 95% CI = 0.568-0.869; P = 0.001). The association was insignificant in adjusted Cox proportional hazards model. The risk of early cessation of EBF was 1.23 times more in those who had not attended PNC than who had attended (HR = 1.229; 95% CI = 1.003-1.505; P = 0.046) and it was 1.85 times more in those who had not received PNC counseling on EBF than who had PNC counseling (HR = 1.854; 95% CI = 1.303-2.640; P = 0.001). Statistical significance was maintained for provision of PNC counseling on EBF in adjusted Cox proportional hazards model and the hazard was 80.8% more in those who did not provided with postnatal counseling on EBF (AHR: 1.808; 95% CI = 1.121-2.914; P = 0.015) and it was insignificant for PNC. First birth order infants were 1.8 times more risk for early cessation of EBF than fifth and higher birth order infants (HR = 1.796; 95% CI = 1.286-2.508; P = 0.001). This was also significant in adjusted Cox proportional hazards model and the hazard of early cessation of EBF in the first birth order infants was 4.72 times that of fifth and higher birth order infants (AHR: 4.720; 95% CI = 1.649-3.511; P = 0.004). Primipara were 1.3 times more risk for early cessation of EBF than multiparae (HR = 1.307; 95% CI = 1.029-1.661; P = 0.028) and mothers who had food insecurity had less risk for early cessation of EBF than those who had no food insecurity (HR = 0.685; 95%CI = 0.553-0.847; P < 0.001). Parity and food insecurity were insignificant in Multivariate Cox proportional hazards model (Table 4).

**Table 4:** **Bivariate and multivariable cox proportional hazards model predicting the hazards of cessation of exclusive breastfeeding by obstetrics and gynecologic variables of mothers of index infants in Ankesha Guagusa Woreda, Awi administrative zone, Northwest Ethiopia 2012**

Variables	Cessation of exclusive breastfeeding			
	CHR^*^(95% CI)	P	AHR ^†^(95% CI)	P
**ANC visit**				
No visit	1.243 (0.658-2.348)	0.503	-------	------
1-3 visits	0.959 (0.784-1.172)	0.683	-------	------
4+ visits	1			
**Counseled on infant feeding during ANC**				
Yes	1		1	
No	1.437 (1.123-1.837)	0.004	1.052 (0.631-1.754)	0.847
**Place of birth**				
Home	0.670 (0.543-.826)	0.000	0.673 (0.344-1.315)	0.246
Health institution	1		1	
**Mode of delivery**				
Vaginal delivery	1		1	
CS	2.543 (1.620-3.993)	0.000	2.103 (1.173-3.770)	0.013
**Birth attendant** Health professional	0.799 (0.549-1.164)	0.243	1.556 (0.785-3.085)	0.205
TTBA	0.555 (0.245-1.257)	0.158	1.240 (0.447-3.442)	0.680
NTTBA	0.703 (0.568-0.869)	0.001	1.040 (0.543-1.990)	0.906
Relatives/friends/neighbors	1		1	
**PNC**				
Yes	1		1	
No	1.229 (1.003-1.505)	0.046	1.947 (0.218-17.391)	0.551
**Counseled on infant feeding during PNC**				
Yes	1		1	
No	1.854 (1.303-2.640)	0.001	1.808 (1.121-2.914)	0.015
**Sex of the index child**				
Male	1		-------	------
Female	1.040 (.853-1.270)	0.697	-------	------
**Birth order of index infant**				
First	1.796 (1.286-2.508)	0.001	4.720 (1.649-3.511)	0.004
Second	1.347 (0.963-1.884)	0.082	1.760 (0.988-3.136)	0.055
Third	1.181 (0.848-1.644)	0.325	1.477 (0.881-2.478)	0.139
Fourth	1.174 (0.811-1.701)	0.395	1.469 (0.855-2.523)	0.164
Fifth and more	1		1	
**Parity**				
Primipara	1.307 (1.029-1.661)	0.028	0.389 (0.146-1.035)	0.059
Multipara	1		1	
**Presence of food insecurity**				
Yes	0.685 (0.553-.847)	0.000	0.677 (0.457-1.004)	0.052
No	1		1	

*Crude Hazard Ratio.

†Adjusted Hazard Ratio.

## Discussion

The factors which affect EBF and duration are not only many and complex, but they work differently in different situations. For example, maternal education has been associated with higher breast-feeding rates in industrialized countries and with lower rates in developing countries. Many other factors affect how women feed their infants and the length of time over which they breast-feed [5, 8, 18–21].

The percentage of infants that stay on EBF for the first 5 months in this study was 63% and this figure is greater than the result of global report (34.8%) and EDHS 2011 report (52%). This percentage increase is consistent with the earlier changes as indicated by the prevalence of EBF was 47% in 2000, 49% in 2005 which gradually increased to 52% in 2011. This is clearly indicative of the presence of improvement in rate of EBF from time to time that may be attributed to the effort of community health extension workers, and is in line with the goal of UN standing committee on nutrition to increase EBF to 6 months of age to a minimum of 60% by 2015 [2, 3, 10, 16].

The life table survival probability distribution indicated that the highest proportion of events before the age of six months was observed during the first month and this result is consistent with the study conducted in Bangladesh in which conditional probability of introducing of complementary foods was highest during the first month [18]. This might be due to the reason that most lactating mothers had begun to engage in usual work after the time of baptism of their child and hence begun to introduce another complementary food to their infants. This finding was also supported by qualitative result in FGD as a 23 years mother stated “*…we are always informed by health extension workers about the time when other foods should be given to our children after 6 months. However, most of us, for instance I gave my infant another food during baptism for the first time to help him take foods later on.”*

The median duration for the entire study subjects in this study was 6.06 months. This was the same with the result of retrospective study conducted in India in which the median duration was 6 months [22]. The median duration of EBF in this was higher in rural (6.36 months) compared to urban (5.13 months) consistent with EDHS 2005 report in which the median duration of EBF was lower in urban residents (1.8 months) compared to rural (2.1 months). However, there is a discrepancy between the figures which is attributed to consideration of censored cases in this study [16].

In this study, the cumulative probability of in taking complementary food at 6 months was 81%. This finding was consistent with the retrospective study conducted in South Gujarat, India [22] and the figure was slightly higher from the study done in Bangladesh (69.9%) and this discrepancy might be attributed to that the study done in Bangladesh was secondary data analysis using Bangladesh Demographic and Health Survey that included children born over the preceding five years [18].

This study attempted to show the factors associated with cessation of EBF in the study area. Among socio-demographic factors included in the study variables, maternal and paternal occupation and place of residence were significantly associated with duration of EBF. Mothers who were engaged in different jobs were 5 times more likely to stop EBF early compared to housewives and this finding is similar with the previous community based cross-sectional study conducted in Adigrat in which mothers working outside home had 3.5 times higher chance of early introduction of complementary food compared to housewives [21]. However, paternal occupation being civil servant was inversely associated as compared to others studies conducted in India [22]. This might be attributed to the fact that civil servant partners were more educated and may know about importance of EBF and gave counseling for their partners. The relative risk of early cessation of EBF was higher in urban residents compared to rural residents (AHR: 2.175) which is similar to the findings of study conducted in Bangladesh, Lebanon and Malaysia [18, 19, 23]. This finding was also supported by qualitative result as a 32 years mother stated “…*most we mothers are working in different works even beginning immediately after a month and mostly 40 days of our delivery, meaning after the date when our child had got Christianity. This resulted to begin feeding of our child with other foods as early as age of 1 month,…for instance I gave cow’s milk for baby by bottle after 1 month”.* Similarly, on another FGD, one respondent from rural residents stated “…*mostly our babies became sick with abdominal cramp when we feed them with breast milk that stayed for a day while we were at work place and market. At that time we give them fresh warmed butter with water to ease from abdominal cramp”.*

Mode of delivery and postnatal counseling on EBF were significant determinants among obstetrical and gynecological factors studied. Mothers who delivered by CS were about twice more risk for early cessation of EBF compared to vaginally delivered mothers and this finding is consistent with the studies done in Eastern Lancashire and Saudi Arabia [24, 25].

Birth order of index infant was significant determinant of cessation of EBF among factors about child characteristics. This study showed that the risk of early cessation of EBF was nearly five folds more in first-born infants than fifth and more born infants. This finding was similar with the reports done in Eastern Lancashire and Brazil [24, 26]. Possible reason for increased hazard of cessation in first-born babies could be that when birth order increases mothers will become more experienced on how to feed their infants. Ethiopian national strategy for IYCF suggested that even though breast-feeding is a natural act, it is also a learned behavior [2].

### Limitations

Since this study was designed to collect data retrospectively, by history, there would be recall bias. But to reduce the recall bias, data were collected by interviewing the mothers to give their response in reference to different events. The most common event used by the respondents as a reference was using the date when their baby became baptized “*Christina*” in Amharic that is practiced by all orthodox Christian followers which took place after 40 days for men and 80 days for women as most of the study subjects were orthodox Christian. Additionally, full month when introduction of complementary feeding is recorded as the time to cessation of EBF in this study than using of the specific date which made the respondents easy to remember that month in reference to different events.

### Strength

Incorporation of both quantitative and qualitative study designs could be considered as the strength. In our country available data on the duration of EBF and its factors were scarce and since this study used Cox proportional hazards model, it is important to get joint estimation of effects of independent variables on the “hazard” (the risk of stopping breast-feeding) than the duration itself and can be used to analyze data that contain censored observations. Since Cox proportional hazards model used partial likelihood function, temporal bias was avoided.

## Conclusion

In this study the highest proportion of introduction of complementary feeding was high during the first month. The median duration of EBF was shorter in urban dwellers than rural dwellers and the difference was statistically significant. It is clear from this study that cessation of EBF was associated with engagement of mothers on jobs related to daily labor, house servant and pottery and their partners who were government employees. In addition, living in urban was significant determinant of cessation of EBF among socio-demographic factors. Among obstetrical and gynecological factors included in the study, giving birth by CS and absence of postnatal counseling on EBF were associated with cessation of EBF. Similarly first birth order infants were at high risk for early cessation of EBF compared to higher birth order infants among biological characteristics of child.

### Recommendation

The findings of this study had its implications for health care workers and other concerned bodies including policy makers to carry out the national strategy on IYCF. The primary focus should be given for the urban dwellers and an effort that could help to let women. An inter-sectoral collaboration, particularly between health care sectors and social and work affairs should be maintained to let those mothers who were engaged in different jobs and there should be child-care facilities, which let working mothers to care for their infants and young children. This will help continued breast-feeding as recommended by global strategy for IYCF to overcome the problem of early initiation of complementary feeding attributed to back to the work. Women in paid employment can be helped to continue breast-feeding by being provided with enabling conditions, such as, paid maternity leave, part-time work arrangements and breast-feeding breaks.

Health professionals who deal with hospital routines and rules should make sure that hospital routines and rules, particularly in case of cesarean section, remain fully supportive of the successful initiation and establishment of breast-feeding and continuation of EBF for the first six months through implementation of the Baby-Friendly Hospital Initiative, monitoring and re-assessing already designated facilities, and expanding the initiative to include clinics, health centers and pediatric hospitals.

Since there is association of cessation of EBF with absence of postnatal counseling on EBF, it is essential to make sure that health worker have updated guidelines, knowledge and skills required to support and counsel all mothers during postnatal period.

Health care workers should make sure that there is an increased advocacy to increase access of all mothers coming for PNC should to postnatal care counseling on EBF counseling. Community health extension workers should bring close monitoring and follow-up care of mothers having infants.

Further research should be done prospectively including knowledge related factors on EBF.
